# Delineation of high resolution climate regions over the Korean Peninsula using machine learning approaches

**DOI:** 10.1371/journal.pone.0223362

**Published:** 2019-10-10

**Authors:** Sumin Park, Haemi Park, Jungho Im, Cheolhee Yoo, Jinyoung Rhee, Byungdoo Lee, ChunGeun Kwon

**Affiliations:** 1 School of Urban and Environmental Engineering, Ulsan National Institute of Science and Technology (UNIST), Ulsan, South Korea; 2 Institute of Industrial Science, The University of Tokyo, Tokyo, Japan; 3 Climate Analytics Department, APEC Climate Center, Busan, South Korea; 4 Forest Conservation Department, National Institute of Forest Science, Seoul, South Korea; George Mason University, UNITED STATES

## Abstract

In this research, climate classification maps over the Korean Peninsula at 1 km resolution were generated using the satellite-based climatic variables of monthly temperature and precipitation based on machine learning approaches. Random forest (RF), artificial neural networks (ANN), k-nearest neighbor (KNN), logistic regression (LR), and support vector machines (SVM) were used to develop models. Training and validation of these models were conducted using *in-situ* observations from the Korea Meteorological Administration (KMA) from 2001 to 2016. The rule of the traditional Köppen-Geiger (K-G) climate classification was used to classify climate regions. The input variables were land surface temperature (LST) of the Moderate Resolution Imaging Spectroradiometer (MODIS), monthly precipitation data from the Tropical Rainfall Measuring Mission (TRMM) 3B43 product, and the Digital Elevation Map (DEM) from the Shuttle Radar Topography Mission (SRTM). The overall accuracy (OA) based on validation data from 2001 to 2016 for all models was high over 95%. DEM and minimum winter temperature were two distinct variables over the study area with particularly high relative importance. ANN produced more realistic spatial distribution of the classified climates despite having a slightly lower OA than the others. The accuracy of the models using high altitudinal *in-situ* data of the Mountain Meteorology Observation System (MMOS) was also assessed. Although the data length of the MMOS data was relatively short (2013 to 2017), it proved that the snowy, dry and cold winter and cool summer class (Dwc) is widely located in the eastern coastal region of South Korea. Temporal shifting of climate was examined through a comparison of climate maps produced by period: from 1950 to 2000, from 1983 to 2000, and from 2001 to 2013. A shrinking trend of snow classes (D) over the Korean Peninsula was clearly observed from the ANN-based climate classification results. Shifting trends of climate with the decrease/increase of snow (D)/temperate (C) classes were clearly shown in the maps produced using the proposed approaches, consistent with the results from the reanalysis data of the Climatic Research Unit (CRU) and Global Precipitation Climatology Centre (GPCC).

## Introduction

Similarity measures of climate between regions normally presented as climate classes are useful for representing spatial environmental characteristics. The changes of climate class boundaries detected by several sets of time-series data from different time periods can reflect climate change [1]. There have been some approaches to regionalize climate areas according to the similarity of regional climatic characteristics in precipitation and temperature schemes based on rule- [2–3], clustering- [4], and machine learning-based classification [5–7].

The rule-based method of Köppen climate classification, which can cover terrestrial regions from tropical to polar areas, has been continuously revised by trial and error since its first publication in 1936 [2–3,8–9]. In particular, the Köppen-Geiger (K-G) climate classification [8] has been regularly used by researchers across a wide range of disciplines for the climatic regionalization of environmental variables, as well as the assessment of the results of climate models, such as the general circulation model (GCM) with multi-scenarios [10–11], hydrological river discharge models [12], and regional climate models [13–14]. A modified version, the Köppen-Trewartha (K-T) climate classification, has been developed with the additional delineation of the high-land class in temperate climate types [3,15]. However, the spatial resolution of the K-G climate classification map of 5 arc-minutes (~0.083 degrees) [16] is higher than that of the most updated K-T reference of 0.5 degrees [15], and the K-G uses more recent periods of climatic datasets compared to the K-T.

Hierarchical and non-hierarchical clustering-based methods, such as single-, average-, complete-linkage, Ward’s method, and k-means clustering, have been developed for grouping a set of objects using the concept of Euclidean distance and defining the climate characteristics of groups. The suitability of a clustering method for climate classification is assessed by comparing the standard deviations within- and between-clusters [4,6].

The number of climate classification studies using different methods has increased in order to avoid the subjectivity that comes from the rule-based method with human experts [7,17]. Some machine learning models have the benefits of automated, hierarchical, and rule-based characteristics of multivariate regression trees (MRT) [7]. Most notably, neural networks have the advantage of handling non-linear relationships among variables through interconnected processing elements [5]. Several machine learning methods in previous climate classification studies—self-organizing map (SOM) in artificial neural network (ANN), decision trees (DT), and MRT—have been used with multiple areas in Puerto Rico, the Carolinas, and even on a global scale, respectively [5–7].

The spatial resolutions of climate models are usually in a range of 50–100 km. Some studies have reported limitations in identifying local climates using these models due to their coarse spatial resolutions, especially in climatologically heterogeneous regions such as mountainous areas including the Alps [9,18–19]. The existing studies have used several interpolation methods to generate higher resolution climate maps. However, due to the complex terrain and the sparse distribution of meteorological data stations, there are uncertainties in East Asia which cause the omission of detailed information when the climate variables are interpolated [20,21]. The Korean Peninsula has also mountainous and rugged terrain in terms of altitudes from the remotely sensed digital elevation maps (DEM). The western and eastern parts of Korean Peninsula show the different variation of temperature clearly. However, such variation is hardly documented in existing coarse resolution reanalysis data, or interpolated data, while it is shown in MODIS LST (1km). In order to resolve uncertainties regarding the coarse resolutions of input data, satellite-based data, higher resolution than the climatic variables data in previous research, can be used as an alternative data source for producing relatively fine resolution climate regions. Rhee et al. [6] developed a climate classification system using a machine learning method with satellite-based variables for North and South Carolina, USA. The research successfully delineated local climates against the National Climatic Data Center (NCDC) climate divisions in the Carolinas, and the quantitative analysis was conducted through comparisons of between- and within-class variations.

Moreover, the finer climate zonation using satellite-based data can give opportunities to explain the details of climate change on a regional scale. The simulation of the shifting of climate classes is one of the main topics in research on the use of the K-G climate classification [1,16,22–23]. In the western US, the disappearance of the alpine tundra climatic type between both durations 1901–1930 and 1987–2006 was discussed in context with the increase of the mean temperature of the warmest month in each elevation [22]. Updating climate zones provides basic and important information for understanding the current stage of climate change. Consequently, remote sensing data are suitable to document the seamless spatiotemporal changes of climatic variables, although they are relatively new and do not cover the historical period of time (e.g., 1950–1970). In addition, one of the merits of using remote sensing data for climate classification is that climate zones in inaccessible areas such as North Korea due to political issues could be delineated using basic meteorological variables with good quality. The Korea Meteorological Administration (KMA) has collected and managed strictly the climatic data of North Korea, which can be used to calibrate and validate climate classification models.

This study adopted supervised machine learning techniques for classification using K-G classes as reference data to train the satellite-derived seamless grid data for enabling effective visualization of climate classes. Sathiaraj et al.[24] used unsupervised clustering methods such as k-means and a balanced iterative reducing and clustering using hierarchies (BIRCH) for climate classification. They pointed out that it is challenging to compare and discuss directly between clustering results and K-G climate maps. They [24] also showed some discrepancies in climate classification results when different periods of input variables were selected such as monthly, annual, and threshold exceeding frequency (TEF). The k-means clustering result with TEF was the case showing higher consistency than the other variables because the thresholds in the K-G method were originally derived by extreme climate cases. Furthermore, there are difficulties in choosing optimal parameters for unsupervised clustering such as the number of seeds, and the class settings regarding how many stations must be included in one class (i.e., they chose 3 stations as minimum for one class). On the other hand, Rhee et al. [6] compared unsupervised and supervised models for climate zone classification. More reliable results were found in the supervised machine learning method when compared to clustering results [6].

In this study, satellite-based data with relatively high resolution are used as input data for empirical modeling with machine learning approaches in order to resolve the uncertainties induced by coarse resolution of the existing climate maps. The objectives of this research are to 1) classify the climate regions over the Korean Peninsula using satellite-based data at 1 km of spatial resolution by machine learning methods and a statistical modeling method, 2) compare the accuracy of the satellite-based climate classification during the 16 years between 2001–2016 with the most recent high resolution global digital map of the rule-based K-G climate classification [16,25], and 3) discuss the trends of climate zone shifting. This study suggests a robust guideline to generate a climate distribution map using satellite-based temperature and precipitation data.

## Materials and methods

### Study area

**Fig 1:**
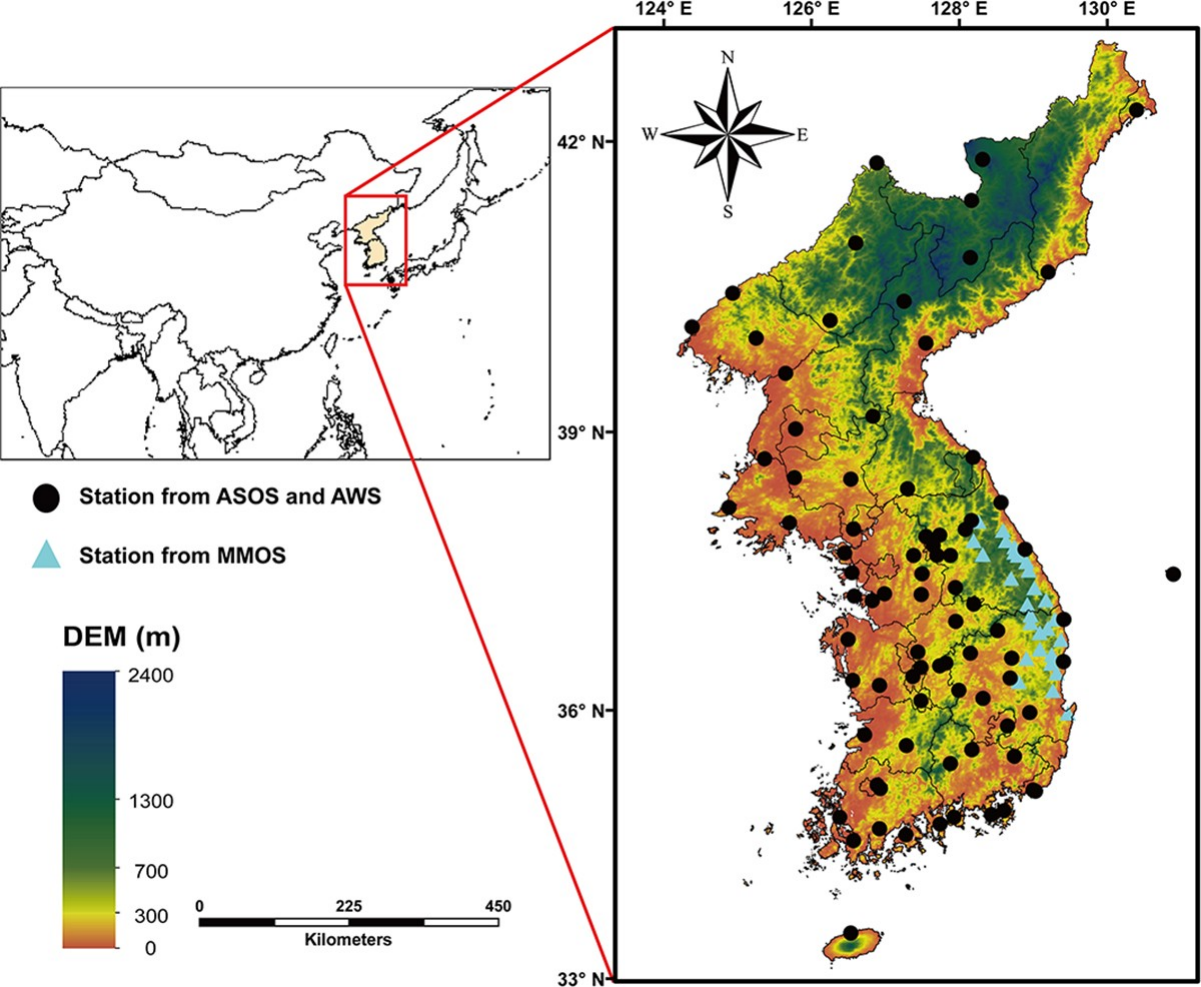
The study area, the Korean Peninsula, including North and South Korea. The Shuttle Radar Topography Mission (SRTM) 30m digital elevation map (DEM; downloaded from http://eros.usgs.gov/elevation-products) is used as a background image.

In this study, the climate zones of the Korean Peninsula including North and South Korea were classified. The Korean Peninsula is located in the range of 33.11–43.01°N latitude and 124.18–131.87°E longitude (Fig 1). The annual average accumulated precipitation and annual average temperature for the study area is 1113.7 mm/yr and 10.52°C/yr, respectively, referring to the meteorological data of KMA during 30 years from 1981 to 2010. The area is normally under the westerly wind; seasonally blowing northwesterly wind from high latitudinal land and southwesterly wind from low latitudinal ocean make the coldest and hottest temperatures in Aug and Jan, respectively. The study area has four distinct seasons, featuring a strong and short rainy season from May to Jun called as “Changma”. Mountainous regions constitute 70% of the terrestrial area according to DEM (Fig 1). As the terrain in the Korean Peninsula is complex and rugged, altitudes drastically differ by administrative district and the patches of land cover are relatively small [26].

### Data

#### Satellite data

The three kinds of satellite data, the Moderate Resolution Imaging Spectroradiometer (MODIS) MOD11A2 V006 Land Surface Temperature (LST), the Tropical Rainfall Measuring Mission (TRMM) 3B43 v7 monthly precipitation, and the Shuttle Radar Topography Mission (SRTM) DEM data, were used for classifying climate zones over the Korean Peninsula. LST was obtained from MODIS on Terra which provides a variety of outputs including land and ocean at 10:30 and 22:30 local time. LST was used instead of air temperature in this study because many researchers have used the LST to estimate the air temperature [27–31]. In this study, the relationship between MODIS LST and *in-situ* air temperature showed a good agreement, resulting in a correlation coefficient (R) of 0.91 and a slope of 1.02, which confirms the effectiveness of using LST as a surrogate variable to air temperature. Monthly information was obtained by converting the MOD11A2 LST considering the number of days. TRMM provides 3B43 monthly precipitation data with 0.25 degree resolution. The product was obtained from the Goddard Earth Sciences Data and Information Center (GES DISC; https://disc.gsfc.nasa.gov/datasets) and resampled to 1km, the same as LST, using a bilinear method. SRTM on the Space Shuttle mission Endeavour STS-9 has C-band Spaceborne Imaging Radar and X-band Synthetic Aperture Radar (X-SAR), and provides DEM data which are produced through the SAR interferometric process. It is provided at 30m and 90m spatial resolution from Elevation Products site of the United States Geological Survey (USGS) (http://eros.usgs.gov/elevation-products). In this study, the 90m resolution was converted to 1km resolution through bilinear resampling for co-locating with LST.

#### Ground meteorological data

In order to develop climate classification models over the Korean Peninsula, ground meteorological data including the Automated Synoptic Observing System (ASOS) and Automated Weather System (AWS) data were obtained from KMA (https://data.kma.go.kr). The data from 2001 to 2016 at 82 stations were used. Data before 2001 were excluded in this study since they were collected from a much smaller number of stations.

The Mountain Meteorology Observation System (MMOS) data have been provided by the National Institute of Forest Science (NIFoS, http://mw.nifos.go.kr). The 15 MMOS stations’ data from 2013 to 2017 were used in this study only in the validation analysis for mountainous areas due to the short period of data collection.

#### Ancillary data

K-G climate classification maps were developed by a rule-based method for classifying climate zones using 50-year air temperature and precipitation reanalysis data. They have been provided as 30 arc-minute (0.5 degree resolution) world maps of KÖPPEN-GEIGER climate classification (http://koeppen-geiger.vu-wien.ac.at/present.htm). There are also mid and high resolution digital maps (10- and 5 arc-minute (~0.17- and 0.083 degree resolution)) obtained by applying the downscaling method introduced by Ruble et al. [16], and using reanalysis data from 1986 to 2010. In this study, we used four digital maps for comparison: two 0.5 degree resolution K-G maps as well as mid and high resolution maps.

Reanalysis data from the Climatic Research Unit Time-Series (CRU TS) 4.01 and Global Precipitation Climatology Centre (GPCC) V7 were used in the K-G climate classification [25,32]. CRU TS 4.01 is produced using angular-distance weighting (ADW) interpolation and provided at 0.5 degree spatial resolution from 1901 to 2016 on the CRU website (https://crudata.uea.ac.uk/cru/data/hrg) [33]. GPCC has been widely used in climate research because it considers the largest number of contributing ground observations [34–35]. For this study, GPCC V7 precipitation data at 0.5 degree spatial resolution from 1983 to 2013 were obtained from the Deutscher Wetterdienst (DWD) (https://opendata.dwd.de/). The K-G climate classification maps based on monthly air temperature from CRU and precipitation from GPCC were derived and used for comparisons.

### Methods

**Fig 2:**
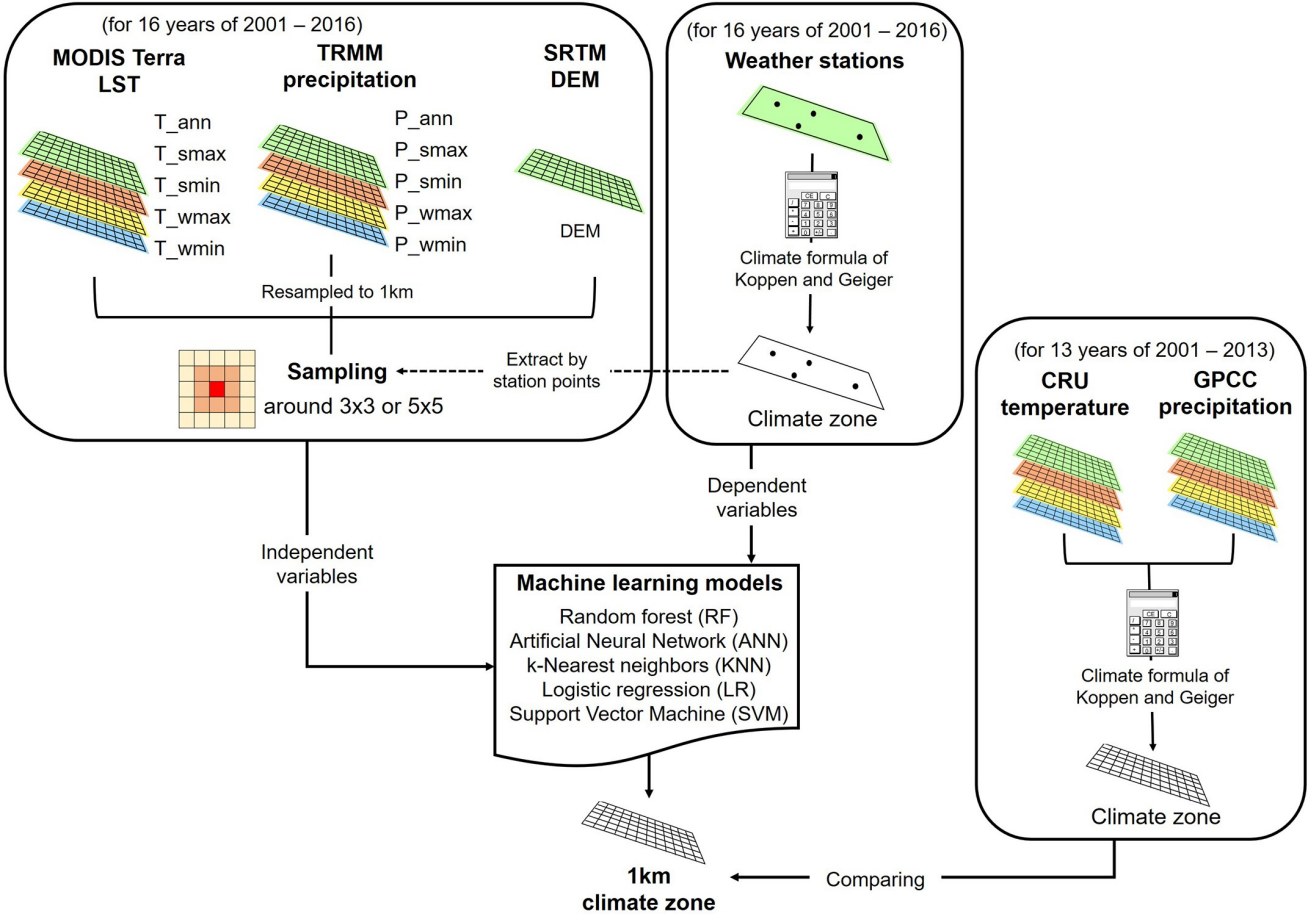
The process flow diagram of the proposed approach for developing climate classification models over the Korean Peninsula.

Fig 2 summarizes how this study was conducted. Firstly, monthly LST and precipitation data from the satellites were used for calculating the following input variables: annual mean LST (T_ann), annual total precipitation (P_ann), and highest and lowest monthly temperature as well as precipitation values for summer and winter (T/P_smax, T/P_smin, T/P_wmax and T/P_wmin). According to the period of ground observations, all satellite data collected from 2001 to 2016 were used for climate classification. Secondly, climate classification classes of 82 stations were calculated using the K-G climate classification formula based on specific thresholds of temperature and precipitation (Table 1). Each station had one class considering climate information from 2001 to 2016. Thirdly, samples were extracted around each station point as the samples for each target class (Table 2). For example, some samples were extracted within a 5×5 window around each station pixel in cases where there were only few stations for certain target classes (e.g., Dwc), while some samples were extracted by an edge of around 3×3 pixels in cases where there were a good number of stations for certain target classes (e.g., Cwa and Dwa) (Table 2). A total of 11 input variables including DEM and the station-based climate class as the target variable were used to develop machine learning-based climate classification models.

**Table 1:** Summary of the K-G formula [25].

Location of character	Type	Description	Criterion
**First**	C	Warm temperate climate	-3°C < Tmin < +18°C
	D	Snow climate	Tmin ≤ -3°C
**Second**	s	With dry summer	Psmin < Pwmin, Pwmax > 3Psmin and Psmin < 40mm
	w	With dry winter	Pwmin < Psmin and Psmax > 10Pwmin
	f	Fully humid	Neither s nor w
**Third**	a	Hot summer	Tmax ≥ 22°C
	b	Warm summer	Not a and at least 4Tmon ≥ 10°C
	c	Cool summer and cold winter	Not b and Tmin > -38°C
	d	Extremely continental	Like c but Tmin ≤ -38°C

**Table 2:** Summary of the reference samples for each target class. The samples were selectively extracted within a window for a station pixel (i.e., pixels in red and light orange color).

	Classes																			
	Cfa					Cwa					Dwa					Dwc				
The number of stations	13					37					35					5				
The sampling method																				
																			
																			
																			
																			
The number of samples	74					118					123					52				

We obtained the following four climate classes over the Korean Peninsula by applying *in-situ* air temperature and precipitation data from meteorological stations to the K-G formula: warm temperate climate with fully humid and hot summer (Cfa), warm temperate climate with dry winter and hot summer (Cwa), snowy climate with dry winter and hot summer (Dwa), and snow climate with dry cold winter and cool summer (Dwc). In order to apply machine learning approaches, the samples were divided into calibration (80%, 257 samples) and validation (20%, 68 samples) and, notably, both datasets were not overlapped. The sizes of input and output data were 257×12 (the number of oversampled stations×the number of input variables) and 257×1 (the number of oversampled stations×climate class) for calibration, respectively. Similarly, the sizes of input and output data were 68×11 and 68×1 for validation, respectively. In order to produce climate zone maps with a size of 1001×1526, 11 input variables were fed into the five models with the size of (1001×1526×11).

#### Machine learning approaches

Machine learning-based modeling has been adopted in many remote sensing applications for both classification and regression [36–44]. In this study, five machine learning approaches—random forest (RF), artificial neural networks (ANN), k-nearest neighbors (KNN), logistic regression (LR), and support vector machines (SVM)—were used for classifying climate zones over the Korean Peninsula.

RF is an ensemble approach based on the Classification and regression trees (CART). It overcomes the limitation of CART such as sensitivity to the training sample and the overfitting problem by aggregating multiple independent trees [45]. RF consists of a variety of classifiers as “trees” which have the same probability distributions from a random selection of training data and input variables through bootstrapping. All trees are aggregated as majority voting for classification or as average for regression. RF provides the relative local importance of input variables which can be obtained as a percentage of the increased mean square error (MSE) through the out-of-bag (OOB) estimate [46–51]. The larger the percentage of increase in the MSE, the more contribution the variable makes to the model compared to others. It should be noted that such importance provided by RF is local, not global though [52–54]. RF package of R-3.6.1 statistic software (http://www.r-project.org) was used with default settings except for the number of trees.

ANN, grounded on the human biological system, is a powerful empirical algorithm for solving nonlinear multivariate problems [55–57]. The basic structural unit is “neuron”, which organizes ANN layers: the input layer, hidden layer(s) and output layer in a typical multi-layer feedforward backpropagation network. All neurons of one layer are connected to the neurons of an adjacent layer using connection weights and biases [58–59]. The training data enter into the input layer as neurons and are multiplied by connection weights and then added/subtracted by biases. The calculated neurons are applied to a transfer function in order to form the neurons of hidden layers. Neurons of output layers are formed in the same way. This process is forward propagation. The error between predicted and true values is fed into updating connection weights and biases through backpropagation. In this study, the “patternnet” function in Matlab R2018a was used for developing the ANN model, which consists of hidden Sizes (structures), trainFcn (learning algorithm) and perfromFcn (loss function). All parameters were selected through many tests with various combinations. ANN structure was organized as one input layer, two hidden layers, and one output layer, where two hidden layers have five and three neurons, respectively. Levenberg-Marquardt (LM) backpropagation was selected as the learning algorithm (called as “trainlm”), which determines the correct combination as minimizing a combination squared errors and weights [60] when updating the weights and biases. Mean squared error (mse) was used for the loss function and default values were used for other parameters (e.g., “tansig” was used for activation function). The best performance was obtained at 25 epochs when the minimum gradient reached.

KNN is one of the widely used nonparametric classification and instance-based approaches, which is based on distance of k nearest sample points (pixels) from an unknown sample point (pixel) [61–65]. It is important to determine the number of nearest points (k). It is sensitive to noise points (overfitting) when k is too small. On the other hand, it can underfit if the k is too large [66]. In this study, the k is optimized by using the “ficknn” function in Matlab R2018a, which automatically finds the number of k and the method of distance measure. The mahalanobis method and one for k were obtained through the optimization.

LR uses the probability of each class to determine the final class for a pixel. In this study, the multinomial logistic regression function (“mnrfit”) in Matlab R2018a was used for developing one of the climate classification models. It uses an iteratively weighted least squared algorithm to find the maximum likelihood estimates [67–68]. For each class, there are coefficients equal to the number of predictors and interception for calculating probability of each class.

As the last method, SVM was used because not only it has been widely applied in remote sensing based classification, but also it has an advantage in working well with the limited number of training data [69–73]. To classify classes, it uses hyperplanes generated by a kernel function. The “fitcsvm” function in Matlab R2018a was used for optimizing the parameters including the kernel function, iteration limit, kernel scale and optimizer for each class. In this study, the polynomial kernel function was used for “Cfa”, “Cwa”, and “Dwa” classes with the polynomial orders of 3, 4, and 3, respectively, and the linear kernel function was used for “Dwc” class. Other parameters were set as 1 for the kernel scale, grid search for the optimizer and 10,000 for the iteration limit.

#### Comparison of model performance with existing climate maps

Three existing climate classification maps were used for comparisons: one map developed by Kottek et al. [25], another map developed by Ruble et al. [16], and a third map calculated by using reanalysis data and machine learning-based models. Kottek et al. [25] used CRU air temperature and GPCC precipitation from 1950 to 2000, which were also used in Rubel et al. [16] as input data for downscaling. The third map was calculated using CRU air temperature and GPCC precipitation through the K-G formula (Table 1) from 2001 to 2013 (due to the limitation of the GPCC period). The use of the existing climate maps is not only to compare with the proposed models for the years from 2001 to 2013, but also to examine climate class change over time divided by two periods (i.e., 2001–2007, 2008–2013).

## Results and discussion

**Fig 3:**
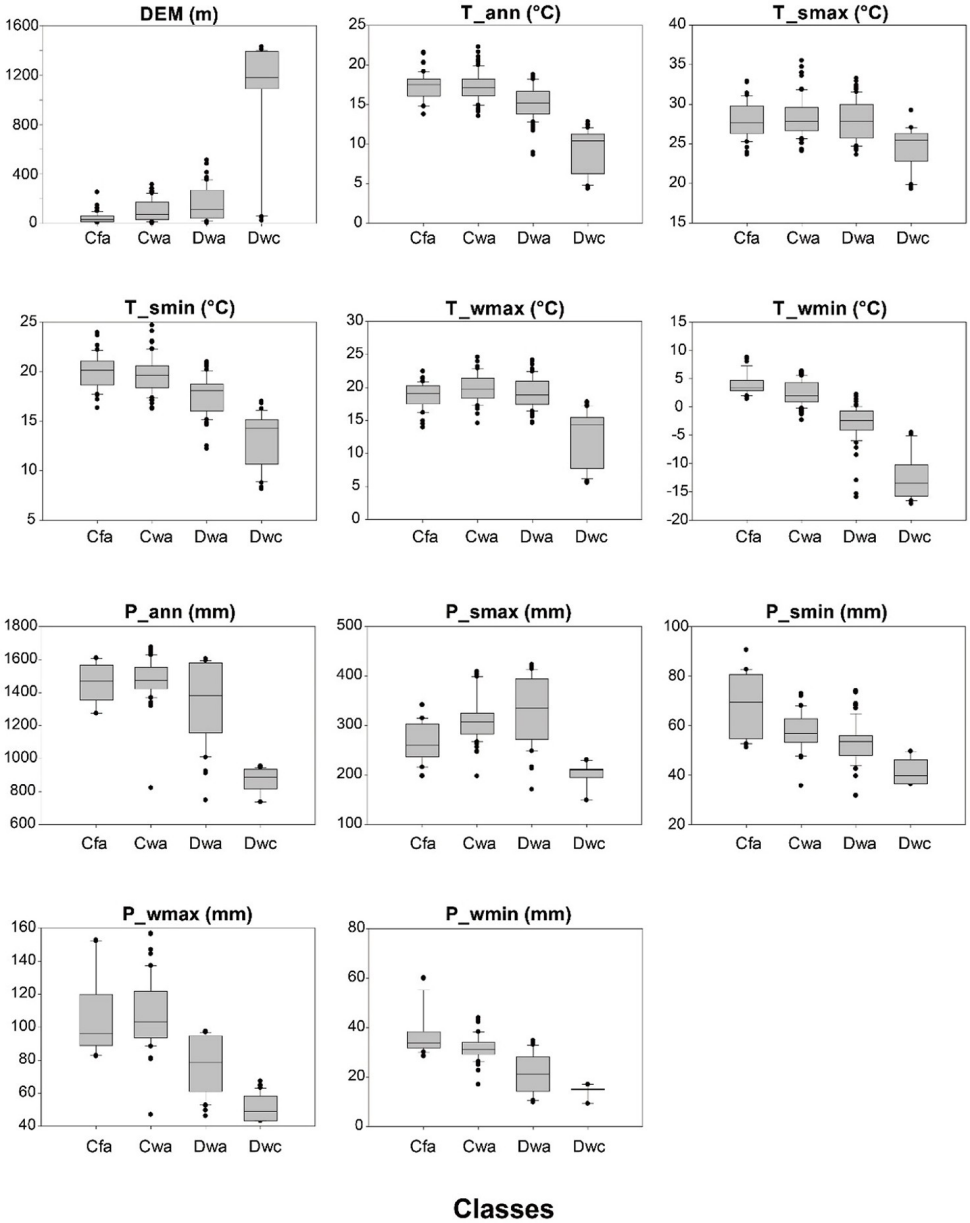
The value ranges of input variables using the calibration data for each target class. Input variables are annual mean LST (T_ann), annual total precipitation (P_ann), and highest and lowest monthly LST and precipitation values for summer and winter (T/P_smax, T/P_smin, T/P_wmax and T/P_wmin), and DEM.

Fig 3 presents the value ranges of input variables using the calibration data set for each target class. Despite using LST instead of air temperature, the value ranges of LST show similar characteristics to those of air temperature according to the K-G formula (Table 1). The Dwc class showed a significant difference in all input variables when compared to the other classes, especially to DEM. When compared with the climate classes that have different first characters of the climate classification (i.e., “C” and “D”), minimum LST and maximum/minimum precipitation for winter (T_wmin, P_wmax and P_wmin) had differences in value ranges consistent with the K-G formula (T min term). When compared with the climate classes that have different second characters of “w” and “f”, the precipitation variables showed larger differences than LST, which is also consistent with the K-G formula (terms of maximum/minimum precipitation for winter and summer). For example, the minimum precipitation for summer of Cfa had relatively high values compared to Cwa, which is similar to the condition of the “f” class in the K-G formula (Table 1). Unlike the K-G formula, not only LST but also precipitation had clear differences when compared to the climate classes that have a different third character (“a” and “c”).

### Climate classification based on spatially distributed data

Table 3 shows the accuracy assessment results of the climate classification maps derived by Kottek et al. (Table 3 (a)) [25], Rubel et al. (Table 3 (b)—(d) [16], CRU and GPCC (Table 3 (e)) and the proposed models (Table 3 (f) and (j)) using the validation data set. While there was no significant difference of accuracy between different periods (i.e., (a), (b), and (e)), there was a difference between different spatial resolutions (i.e., (b)—(d)). The proposed models show the best level of accuracy as shown in Table 3, with 100% and 97.06% for validation of RF (f), KNN and SVM (h and i), respectively. Accuracies of ANN and LR were 95.6%. All of the proposed models in this study showed higher performance than the existing models in the accuracy of climate zone classification. The reason that the existing models ((a)–(e)) have lower accuracy is possibly due to the coarse spatial resolution and uncertainty of reanalysis data. The difference in monthly air temperature and precipitation were about 1.6°C and 50mm respectively over the Korean Peninsula. Most notably, it is difficult to apply the K-G formula directly to the reanalysis data since the air temperature difference is above 2°C and precipitation difference is above 50mm due to the non-matched stations between reanalysis data and *in-situ* observation-based climate classes (i.e., 22 stations). The proposed models, on the other hand, did not have such a problem in applying the reanalysis data to the simple thresholds because they were developed using machine learning approaches targeting *in-situ* data.

**Table 3:** Accuracy of climate classifications by model based on the validation data.

**Model**	(a)	(b)	(c)	(d)	(e)
	Kottek et al. (0.5°)	Rubel et al. (0.5°)	Rubel et al. (0.17°)	Rubel et al. (0.08°)	CRU&GPCC (0.5°)
**Accuracy (%)**	58.82	57.35	69.11	69.11	55.88
**Model**	(f)	(g)	(h)	(i)	(j)
	RF. (0.01°)	ANN (0.01°)	KNN (0.01°)	LR (0.01°)	SVM (0.01°)
**Accuracy (%)**	100	95.6	97.06	95.6	97.06

**Fig 4:**
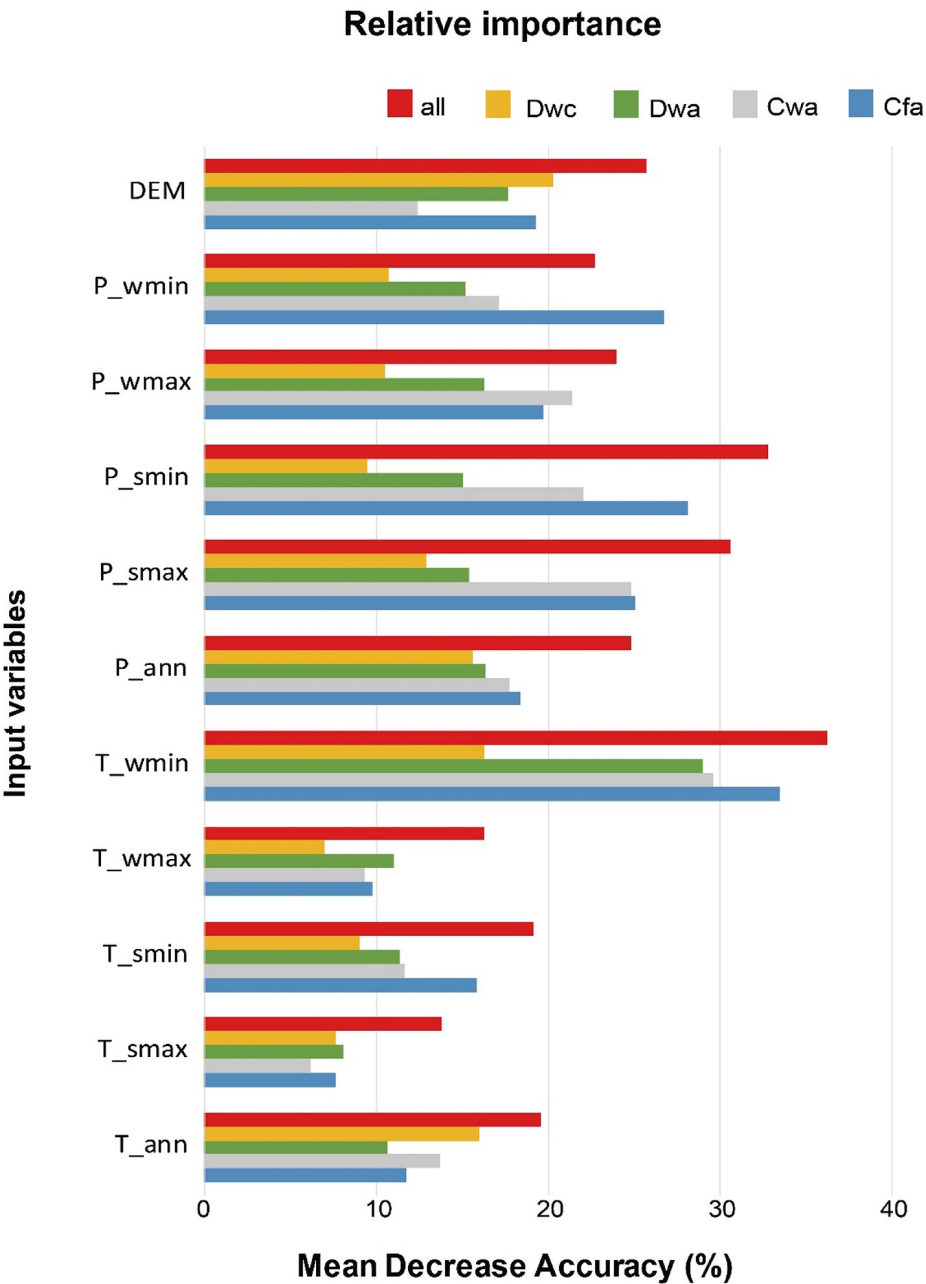
The relative importance (%) of input variables (annual mean LST (T_ann), annual total precipitation (P_ann), and highest and lowest monthly LST and precipitation values for summer and winter (T/P_smax, T/P_smin, T/P_wmax and T/P_wmin), and DEM) using random forest (RF).

Fig 4 shows relative variable importance for observing an overall trend; each climate class is shown in terms of mean decrease accuracy (MDA), which is a widely used measure for regression as well as classification in RF [74–78]. As observed in the differences of the value ranges of input variables for each class in Fig 3, the minimum LST for winter (T_wmin) was the most contributing variable in the RF model (Fig 4, red boxes). It is consistent with the fact that air temperature plays an important role in classifying either warm temperature or a snow climate in the K-G formula (Table 1). Except for T_wmin, precipitation terms had a larger relative importance than temperature terms, because the difference in the range of precipitation values of the four classes was remarkable when compared to temperature variables in Fig 3. For example, T_smax did not show a significant difference in each class (Fig 3) and all classes showed low importance (Fig 4). The reason why DEM has the highest MDA is because the Dwc class has a range that is significantly different from other classes (i.e., high altitude (Fig 4, Dwc station)).

**Fig 5:**
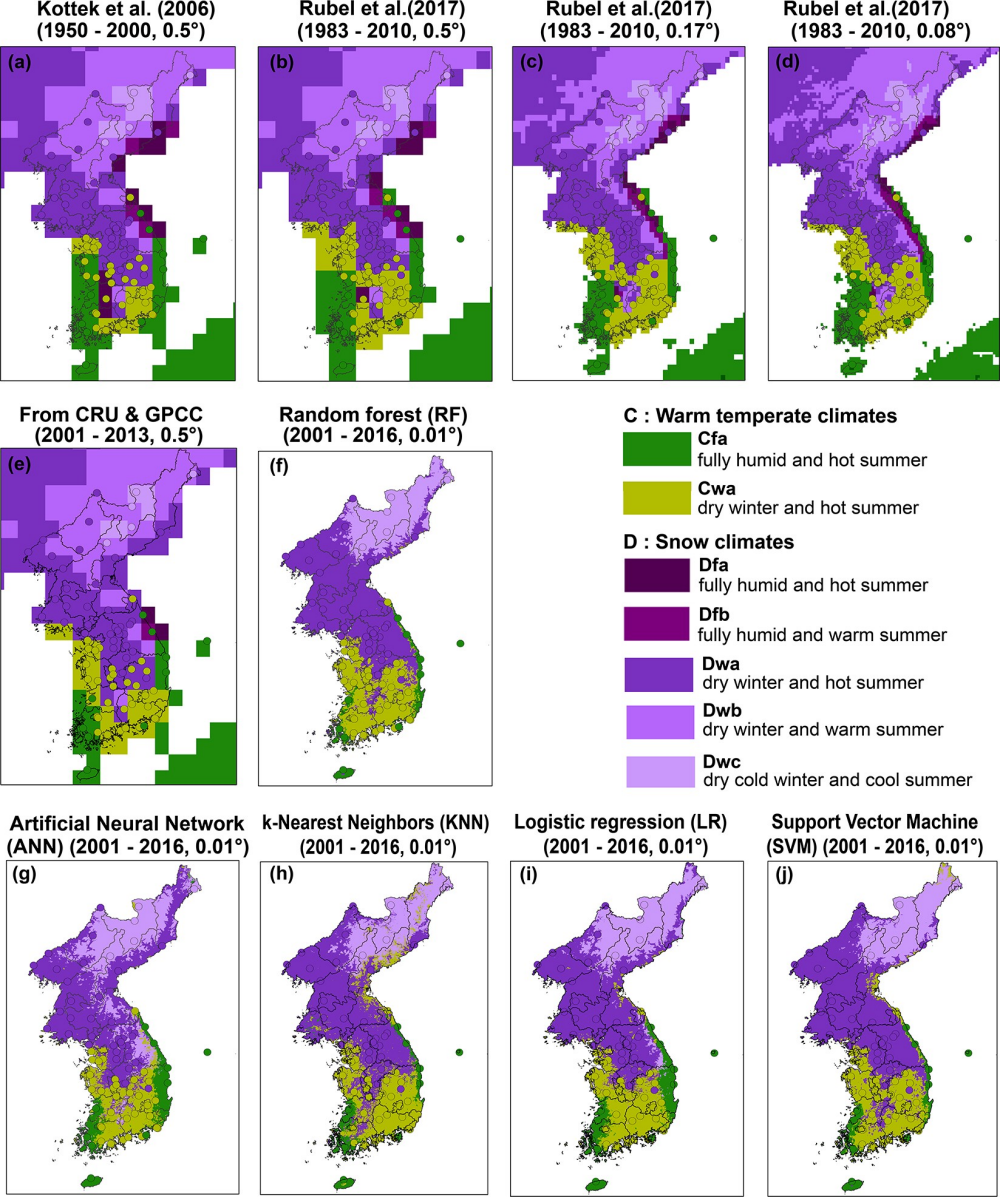
Reference data from *in-situ* weather stations of Automated Surface Observing System (ASOS) and Automatic Weather System (AWS) with circle symbols on the climate classification maps derived by (a) Kottek et al. [25], (b-d) Rubel et al. [16], (e) the calculation based on the CRU temperature and GPCC precipitation, (f) random forest, (g) artificial neural network, (h) k-nearest neighbors, (i) logistic regression, and (j) support vector machine models.

The distribution of climate classification over the Korean Peninsula is described from the outputs of previous studies (Fig 5A–5E) and this study (Fig 5F–5J). All maps (including grids and circles) have similar distributions as warm temperate climates over the southern area of the Korean Peninsula, and snow climates over the northern area and areas of high elevation (refer to DEM distribution in Fig 1). Fig 5A is based on the Kottek et al. [25] map, which used CRU air temperature and GPCC precipitation from 1950 to 2000 while Fig 5B is based on the data from 1983 to 2010. Some changes are observed depending on the applied period; the area of Cfa on the coast decreased in size while the area of Cwa inland increased over time. However, there are some non-matched stations on both 5a and 5b, especially on the coastal line and in relatively high elevation areas, due to the error in the climatic data as mentioned in Table 3 (1.6°C and 50 mm/month of errors from *in-situ* data). The discrepancies between the gridded climate variables and *in-situ* measurements in highly elevated regions are known to be larger than those in flat regions [79]. Usually, the large biases of climatic data in highly elevated regions and coastal regions are challenging issues for accurate interpolation because of the relatively sparse distributions of weather stations to be used in distance-weighted methods [80]. Maps with finer spatial resolutions (Fig 5C and 5D) show more matching classes between the pixels and stations both on the east coast and in the southern inland than the maps with coarser resolutions (Fig 5A and 5B).

In order to compare the maps produced by the proposed models with those from previous studies, we classified climate regions using CRU air temperature and GPCC precipitation data based on Table 1 (Fig 5E). Although the spatial distribution of Fig 5E is consistent with previous studies, Cwa pixels in South Korea are classified as Dwa. Results from the proposed models (Fig 5F–5J) have similar distributions to results from the other models (from Fig 5A–5E). For example, the distributions of warm temperature (C) and snow (D) climates are similar in the coastal area. The effects of complex terrain also appear well in the results from the proposed models, for example near the central region of South Korea with Dfx and Dwx classes. However, the results from RF, KNN, and SVM (Fig 5F, 5H and 5J), and those from ANN and LR (Fig 5G and 5I) yielded somewhat different distributions of classes, especially for Cfa and Dwc. Although the accuracy of RF is 100% using the validation data set, the results showed larger differences from surrounding pixels and the elevation effect was not captured well. On the other hand, the climate regions from ANN were well distributed including coastal lines and the elevation effect was well reflected, through visual inspection of the results. The LR model following ANN well detected the hot and warm summer distributions between west and east of South Korea along the high altitudinal area. It is known that supervised-machine learning classifiers tend to be confused, particularly in the case of mixed classes that share a similar variability of features [58,81–83]. The accuracy may vary depending on the type of the model used [84]. RF showed more confusion than ANN, resulting in merged large areas where similar classes are located (Fig 5F and 5G). For example, if there are boundaries between Cfa and Cwa or between Dwa and Dwc, RF tends to be biased towards more widely (dominantly) distributed classes, Cwa or Dwa, respectively. In other words, ANN delineated the spatial representativeness of climate regions well in comparison to the references from *in-situ* stations and DEM. ANN showed better performance than RF in terms of the spatial distribution of climate classes. Furthermore, KNN and SVM results showed some warm classes (Cwa) in the coastal area of North Korea. In particular, the map of KNN model has salt-and-pepper noise in some areas because of small k-value (k = 1) [62, 66]. Since there are no stations where exactly overlap with grids, the accuracy should be discussed by using ancillary data such as MMOS.

### Evaluation of climate classification based on MMOS

**Fig 6:**
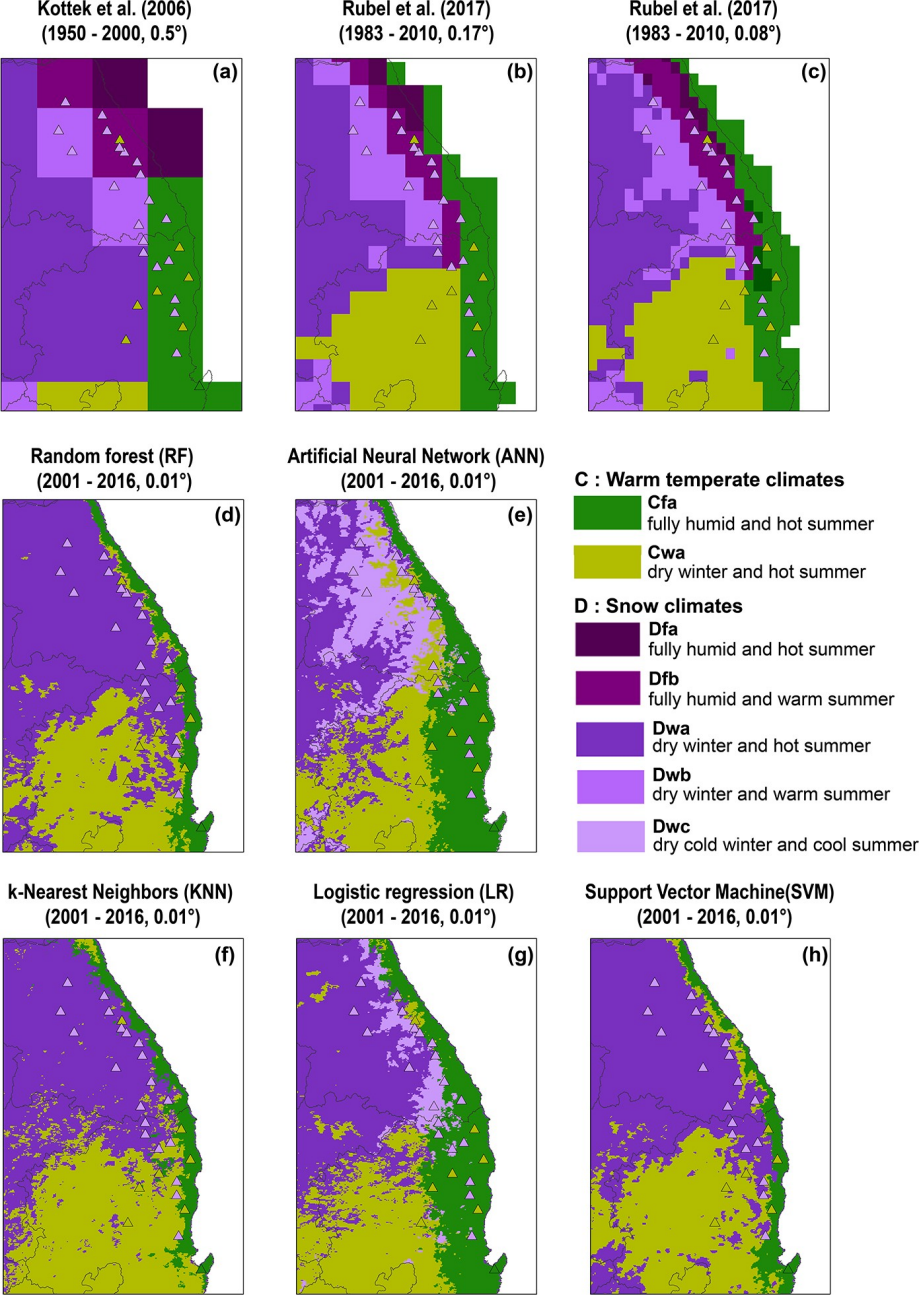
Short-term (2013–2017) evaluation of Köppen-Geiger climate classification using *in-situ* data from Mountain Meteorology Observation System (MMOS) stations with triangular symbols on the climate maps which are derived by a) Kottek et al. [25], b) and c) Rubel et al. [16], and d) random forest, e) artificial neural network, f) k-nearest neighbors, g) logistic regression, and h) support vector machine models.

For a further analysis focusing on mountainous areas in South Korea, MMOS data from 2013 to 2017 were applied to the classification maps (Fig 6). One issue is the non-existence of snow and fully humid classes of Dfa and Dfb from *in-situ* observations of KMA ASOS used in this study, since those classes are included in Kottek et al. [25] and Rubel et al. [16]. Denser but shorter-term *in-situ* reference data (2013–2017) from MMOS stations were used to examine whether or not the high altitudinal mountainous area shows Dfa/Dfb classes (Fig 6). However, neither Dfa nor Dfb appeared from the rule-based K-G calculation based on MMOS. Although the training data used for proposed models (Fig 6D–6H) were from a different data source (i.e., ASOS), the results show similar patterns with those of the MMOS references (triangular symbols) in the locations of the boundary lines of the climate zones. The accuracy in percent when the spatial distributions of climate classes were validated by 15 MMOS station data is shown in Table 4. The ANN model produced the best performance (57.14%) while the coarser resolution maps had much lower accuracy (10.71–3.57%). The range of accuracies of the other models showed slightly higher than the existing maps (32.14–10.71%). Interestingly, the result of Rubel et al. [16] at 0.08 degree resolution was less accurate than that at 0.17 degree resolution. One of the possible reasons is that the interpolation used in Rubel et al. [16] to generate different scales of climate maps was not sufficient to explain the climate distribution in eastern Korea at 0.08 degree spatial resolution. Original temperature and precipitation data from CRU and GPCC respectively in Rubel et al. [16] have coarse resolution before resampling. They thus used DEM and the lapse rate of temperature decrease for modifying the climatic variables considering the topographic effect using the Global 30 arc-second elevation (GTOPO30) of the U.S. Geological Survey (USGS). It seems that data at 0.5 degree resolution could be effectively resampled to 0.17 degree resolution (Table 4). However, the accuracy of the climate map at 0.08 degree resolution (Table 4) showed no improvement when compared to the map at slightly coarser 0.17 degree resolution.

**Table 4:** Accuracy of climate classification on Mountain Meteorology Observation System (MMOS) stations.

**Model**	Kottek et al. (0.5°)	Rubel et al. (0.5°)		Rubel et al. (0.17°)	Rubel et al. (0.08°)
**Accuracy (%)**	3.57	7.14		14.29	10.71
**Model**	RF (0.01°)	ANN (0.01°)	KNN (0.01°)	LR (0.01°)	SVM (0.01°)
**Accuracy (%)**	10.71	57.14	14.29	32.14	10.71

The relief of the eastern Korea mountainous Taebaeksan Range is a typical steep region that faces a number of landslide events [85–86]. The topographical effect to climatic variables, especially wind speed, is clearly shown by classifying geomorphological characteristics into pass, peak, and ridge in Gangwon Province of the eastern Korea [87]. Rubel et al. [16] also mentioned the possible existence of a source of uncertainty due to local effects, including the rain shadow, especially in the Alps region. Hence, we carefully analyzed the differences in spatial distributions with the valid data set measured over mountainous areas (i.e., MMOS). The most current *in-situ* data (i.e., MMOS) shows a decrease of areas of Cfa (Fig 6). The trend of decreasing Cfa is reflected in the shifting of climate zones (Fig 5) as well.

In terms of a spatial resolution, the maps with 0.17 and 0.08 degree spatial resolution showed slightly different distributions: 1) The finer resolution map produced more Cfa class areas, 2) scattered pixels with “D” classes were found within the Cwa area, and 3) details of changes of climate zones showed similar pattern to DEM. The most well-matched climate map with the MMOS *in-situ* reference data is the result of ANN at 0.01 degree spatial resolution (Fig 6D). However, the unrealistic pattern of Dwa scattered within the Cwa area still remains as a limitation of the proposed models due to the relatively small number of training samples to cover the entire study area. It might be due to the possible overfitting of the ANN model with a complex structure, trained even with outliers [88].

### Climate zone shifting

**Fig 7:**
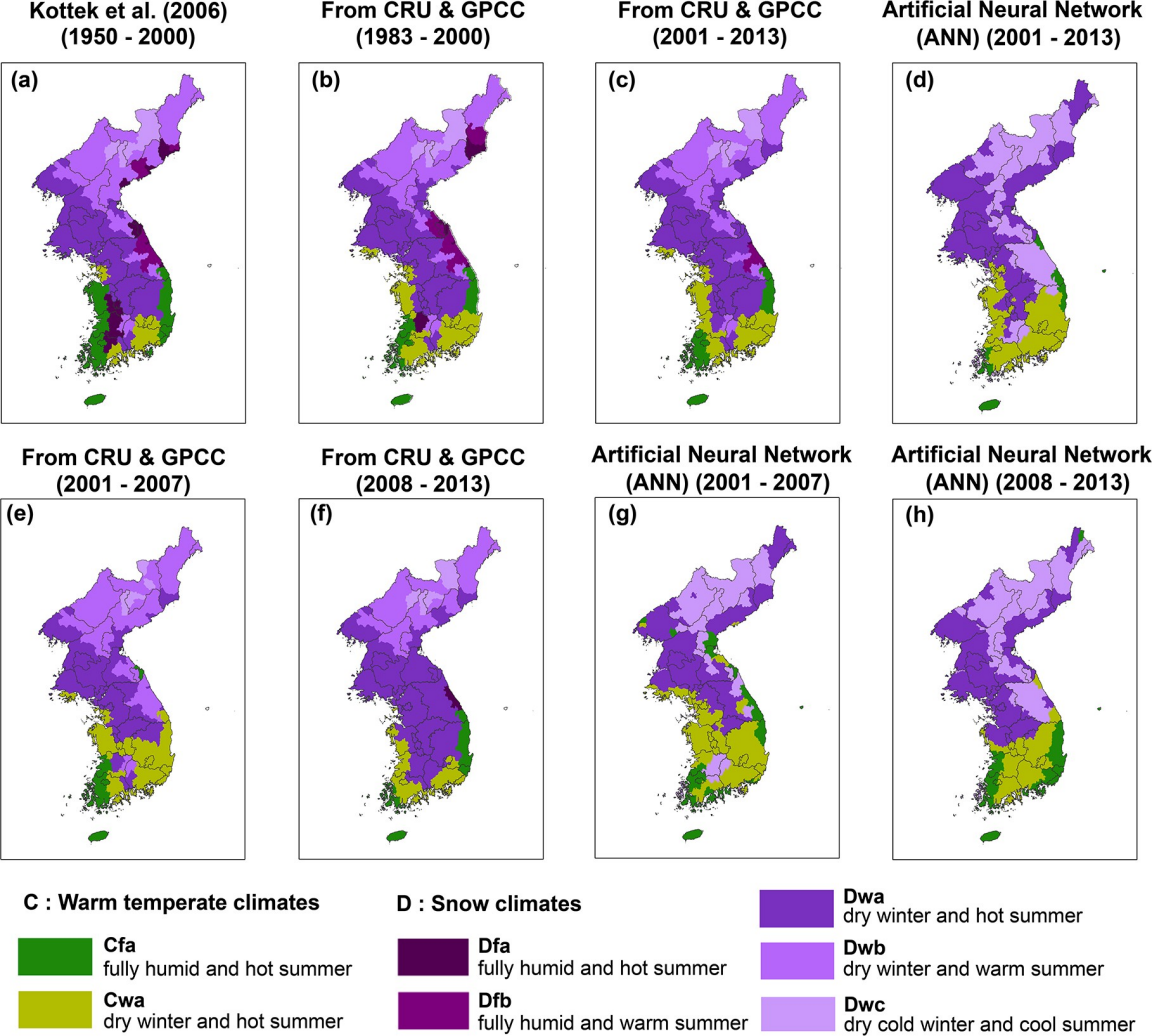
Zonal mean maps of Köppen-Geiger climate classification based on administrative districts of Korean Peninsula. (a) Reference map of Kottek et al. [25] for 1960–2000. Climate classifications using CRU temperature and GPCC precipitation (b) for 1983–2000, and (c) for 2001–2013. (d) climate classification using satellite-based land surface temperature and precipitation with the ANN approach. (e)-(h) Short-term changes from 2001–2007 and 2008–2013 using CRU and GPCC, and using satellite-based data with ANN, respectively.

Climate maps were derived by several different time-series of data for describing climate zone shifting over time; the decadal changes can be found in Fig 7. It should be noted that ten years of data may not be sufficient for determining climate zones. However, they can roughly display a changing trend in climate zones over time. Three maps (Fig 7A–7C, 7E and 7F) were generated by the rule-based K-G method according to the conditions in Table 1. Fig 7D, 7G and 7H depict the result of the ANN model that has relatively good performance in mountainous areas in this study by training satellite-based meteorological data targeting *in-situ* observations. All maps were zonally averaged using a zonal mean function in ArcGIS for administrative districts of South and North Korea. For temperate climates (“C”), high and low humidity of the winter season determines Cfa and Cwa, respectively. In addition, the shrinking trend of Cfa in the western part of the study area is well documented from 1950–2000 to 1983–2000.

The changing trend from Cfa to Cwa is stronger between the rule-based map (Fig 7B) and the ANN map (Fig 7D) than between the two rule-based maps for the period of 1983–2000 (Fig 7B) and 2001–2013 (Fig 7C). The difference between Cfa and Cwa is in the second letter; “f” is for fully humid and “w” is for dry winter. If precipitation (P) is concentrated in summer and the amount is the tenfold of winter P, the second letter of the climate class should be “w”. Since the “C” classes appeared in coastal regions, the shrinking trend in Cfa areas means that the humidity of the south-west and south-east coastal regions of the study area is decreasing. The smallest area of Cfa was found in Fig 7D, which used satellite-based data as input. Moreover, many districts showed changes to the dry winter class while temperature in summer was still classified as hot summer. This might be due to the discrepancy between the TRMM monthly precipitation data and the KMA *in-situ* data; the RMSE and R^2^ between the two sources were 52.88 (mm/month) and 0.84, respectively.

In terms of the shifting of cold climate zones, the southern borderline of Dfa moved northward and its area shrank. The decreasing pattern of the “D” classes is also found in an existing study using the Trewartha climate classification [89]. In Yun et al. [89], the boundary lines of subtropical climate in present, near-future, and future cases also moved northward. The shifting of “D” over time might be related to the effect of global warming on the increment of temperature in the Korean Peninsula. Snow climates denoted as “D” are located along the mountainous areas with high elevation (Figs 1 and 7). Although the ANN model in this study was trained using *in-situ* references of four classes without Dfa, Dfb, and Dwb, the distribution of the cold class (Dwc) is well matched with the high altitude areas of DEM (Fig 1).

Changed areas from Dfb to Dwb can be found in the comparison of the maps using data from 1983–2000 to 2001–2013 in Fig 7. The trend that the fully humid class decreases is clearly shown, not only in cold (“D”) but also in temperate (“C”) classes. Humidity, which is described using precipitation in the K-G climate classification, would also be affected by temperature. The disappearing pattern of “D” classes is also clearly described in the global present and future climate classification maps at 1-km resolution comparing two periods from 1980 to 2016 and from 2071 to 2100 in Beck et al. [90]. Moreover, the high uncertainty, which remains around the coastal regions of the Korean Peninsula, was found in their analysis of confidence levels (%) derived from 12 kinds of different combinations of climate datasets. This implies that the results of climate classification strongly depend on the input datasets used for classifying climate because the number of stations, the durations of obtained datasets, and the resolution for interpolation are different. Furthermore, the discrepancy in the results of the confidence levels when various combinations of input datasets were used in [90] was relatively high in Korea. In that sense, the high accuracy of climate classification methods proposed in this study, which is trained and validated by intensively obtained *in-situ* observation data, can contribute to provide new insight on a region that has thus far suffered from a lack of confident climate classification.

Fig 7 documents the temporal shifting of climate characteristics at a small (6 years) scale. Regardless of modeling methods used, the changes of detailed climate characteristics in 6 years were found in the southern part of the Peninsula. As the climate is determined by several decadal periods [91], it is hard to say that the change in the classification maps is the change of climate. However, the climate similarity is different between the warm temperate and snow climates (Table 5). The area where D classes were converted to C classes using ANN between the periods 2001–2007 and 2008–2013 was 10.1% (14,008km^2^). In contrast, the area converted from C to D classes was 36.97% (51,290km^2^) of all changed areas.

**Table 5:** The numbers of pixels in the climate classes shifted from 2001–2007 to 2008–2013 using the ANN model.

	ANN from 2008 to 2013				
**ANN from 2001 to 2007**		**Cfa**	**Cwa**	**Dwa**	**Dwc**
	**Cfa**		7931	10487	1186
	**Cwa**	20440		36911	2706
	**Dwa**	1166			40935
	**Dwc**	452	12390	4122	

In the perspective of the humidity, the areas converted from Cfa to Cwa and vice versa were 5.72% (7,931km^2^) and 14.73% (20,440km^2^), respectively, from 2001–2007 to 2008–2013. Larger area was converted from dry (w) to humid (f) than from f to w during the periods. The areas converted from w to f, and from f to w were mainly distributed in the southern and northern parts, respectively (Fig 7). There are previous studies about decadal changes in climates regarding the summer precipitation and humidity [92, 93]. In particular, the summer precipitation was revealed to increase in terms of amount and intensity from 1973 to 2005 in South Korea [92]. As an example of the regional changes of climate regarding humidity, the decadal changes in the boundary of humid and semi-arid regions were explicitly depicted in China [93]. Our result of climate shifting for 12 years also showed changes in areas, which might correspond to those phenomena in East Asia [92,93]. In the summer temperature scheme, the areas converted from Dwc to Dwa and vice versa were 2.97% (4,122km^2^) and 29.5% (40,935km^2^), respectively. There were clear trends from dry to humid and cool to hot summers between 2001–2007 and 2008–2013. The results imply:1) the southern boundary of D class moved towards the northern area of Korean Peninsula for long-term change (1983–2013), 2) the northern and the mountainous parts of Korean Peninsula could be cooler and more humid than before for short-term change (between 2001–2007 and 2008–2013) since those areas were converted to D and w from C and f in terms of temperature and humidity schemes, respectively. It should be noted that longer temporal accumulation of data is crucial for the delineation of climate in global scale since short-term averages are changeable and unstable when compared to long-term averages [91].

Past studies agree with that the climate zone in term of temperature is shifting to warmer [32, 94, 95]. Rubel et al. [32] simulated global climate using the GCM and K-G method in both periods 1901–1925 and 2076–2100, and found that most countries in mid-latitudes including Korean Peninsula would become warmer. Although the changes in all climate zones were not clear from their results due to the coarse resolution (50km), the trend of climate zone shifting from D to C was clearly identified [32]. Furthermore, the effect of shifting to warmer classes is becoming rapid especially in mountainous area from the analysis of vegetation habitat and glacial coverage [16, 94]. Our results in the long-term change (from 1983–2000 to 2001–2013) of climate zone also show a warming trend, which is consistent with global climate shifting simulations.

## Conclusions

In this study, fine spatial resolution (1km) climate classification maps were developed using machine learning approaches (ANN, KNN, RF, LR, and SVM) targeting *in-situ* data over the Korean Peninsula. This study proves not only the applicability of machine learning approaches, but also the usability of satellite-based precipitation data and surface temperature data for classifying climate zones. Both precipitation and temperature showed a distinct range of variations by climate class. In particular, the input variables (i.e., P_smin and T_wmin) that are prioritized in the K-G formula showed large differences of variations by class and high variable importance. Including the machine learning based maps, all climate classification maps have similar distribution of snow climates (northern area) and warm temperate climates (southern area). The maps produced using the proposed models showed better accuracy when compared to the existing maps. In particular, the ANN result showed better performance than KNN, LR, RF, and SVM when considering the spatial distribution of *in-situ* observations (i.e., ASOS, AWS and MMOS) and elevation data. Long-term time-series analysis based on the ANN results showed the decrease in snow climate areas (D) and increase in warm temperate climate areas (C) in the Korean Peninsula. Short-term time-series showed an inconsistent result when compared to the long-term results. However, conspicuous changes in climate classes were confirmed from the short-term analysis. The proposed methods can be used as a robust guideline to generate climate classification by applying satellite-based temperature and precipitation data.

However, there are some limitations, including the relatively small number of samples and the short coverage period of MMOS data, which deserve for future research. The number of climate class types and samples can increase through extending the study area to cover more of East Asia. In addition, additional input data, such as soil moisture, evapotranspiration and landcover may improve the performance of the proposed approaches for climate classification in the future work.

## Supporting information

S1 Dataset Calibration and validation data used to develop machine learning models (the first row is variable names) and input variables for generating maps from 2001 to 2016. (ZIP)

S1 Model Model files developed in this study: artificial neural network, k-nearest neighbors, logistic regression, random forest, and support vector machine. (ZIP)
